# Deep Anterior Lamellar Keratoplasty Over Penetrating Keratoplasty to Mitigate Positive Vitreous Pressure Complications

**DOI:** 10.7759/cureus.51120

**Published:** 2023-12-26

**Authors:** Khaled Moumneh, Scott Pollack, Larry M Perich

**Affiliations:** 1 Ophthalmology, HCA Florida Bayonet Point Hospital, Hudson, USA; 2 Ophthalmology, Pollack Eye Center, Buffalo, USA; 3 Ophthalmology, Perich Eye Center, New Port Richey, USA

**Keywords:** donor cornea, combined surgical technique, deep anterior lamellar keratoplasty, cornea transplant, ophthalmology

## Abstract

This report describes deep anterior lamellar keratoplasty over penetrating keratoplasty (DALK-over-PKP) as an alternative technique to mitigate complications related to positive vitreous pressure (PVP) during PKP. We accomplished this by repairing the punctured cornea and performing a modified DALK where a full-thickness donor graft is placed over the host Descemet membrane, which is then removed after partial suturing of the graft. This mitigates the driving force behind the PVP by maintaining a closed-anterior chamber.

## Introduction

Penetrating keratoplasty (PKP) is a technique that involves the complete removal of all layers of the central cornea followed by replacement with a donor cornea, which is sutured into place. Indications for PKP include keratoconus, corneal dystrophies, viral keratitis, mechanical trauma, and other full-thickness corneal pathologies [1]. Deep anterior lamellar keratoplasty (DALK) is a procedure involving a partial-thickness corneal transplant leaving the host Descemet membrane and endothelium intact. This technique evolved from lamellar keratoplasty in 1984 as an alternative to the full-thickness corneal transplant of PKP. DALK is typically performed via trephination of the host cornea two-thirds of the way through the stroma, followed by dissection of the stromal layers above the Descemet membrane using a blade. Indications for DALK include healed infectious keratitis-induced blindness, keratoconus, post-refractive surgical keratectasia, corneal stroma dystrophies, corneal clouding in mucopolysaccharidosis, superficial corneal scarring, perforations, and other superficial pathologies [2].

To better visualize depth during dissection, prevent complications related to perforation, and increase overall success of the procedure, numerous strategies to perform DALK have been developed. The “big-bubble technique” involves injecting a large bubble after trephination, which creates a bulge in the Descemet membrane, allowing easier separation and dissection of the layer. The depth of the dissector during a manual dissection can be better visualized by injecting air into the anterior chamber creating a mirror [3]. Hydrodelamination can be done by injecting a balanced salt solution into the stroma, causing swelling and allowing manipulation via forceps [4]. More recently, automated approaches have been developed using an automated microkeratome or a femtosecond laser, which will precisely cut at a specific depth [5,6]. Comparisons between DALK and PKP have shown an advantage anatomically in terms of increased endothelial cell count long term in DALK; however, this does not translate functionally as there is no statistical advantage in long-term best-corrected visual acuity [7]. Today, DALK is often used in lieu of PKP due to the many other advantages it affords. Common complications of PKP when compared to DALK include a higher graft rejection rate, slow healing, and potential for ocular structural damage due to the “open-sky” nature of the surgery. On the other hand, DALK has a higher likelihood of causing induced astigmatism due to the potential uneven dissection and intraoperative surgical failure depending on the skill level and comfort of the surgeon [8]. Despite the safety and strength of DALK and other closed-system corneal procedures, PKP remains necessary especially in cases without an intact endothelium or the Descemet membrane, prior ocular surgeries, and full-thickness corneal damage caused by trauma or other pathologies [9].

In anterior segment intraocular surgery where a full thickness incision to the cornea is created, such as in PKP or cataract extraction, a common complication is positive vitreous pressure (PVP), which occurs secondary to a variety of mechanisms [10]. Acute hypotony is caused by an open anterior segment and subsequent aqueous loss leading to a pressure gradient and elevated vitreous pressure [11]. External compression of the globe either with a tight lid speculum or anything that causes increased orbital pressure, such as retrobulbar block or hemorrhage, can lead to vitreous cavity volume reduction [12,13]. Intraocular intumescence can cause vitreous volume reduction due to mechanisms, such as congested choroid from congestive heart failure (CHF) or obesity and intraoperative choroidal hemorrhage [14,15]. The end result is anterior displacement of the lens-iris diaphragm, leading to iris prolapse, flattening of the anterior chamber, anterior lens prolapse, vitreous prolapse, and zonular rupture, thus increasing the complexity and risk of the surgery [13].

PVP risk can be decreased by minimizing the degree and duration of ocular hypotony during surgery by maintaining aqueous pressure via viscoelastic or air bubble. Systemic risk factors, such as hypertension, CHF, preoperative ocular hypertension, and preoperative choroidal effusion, should ideally be controlled. During the surgery, the pressure of the lid speculum, lids, and instruments on the globe should be minimized. Following administration of a retrobulbar block, IOP can be lowered preoperatively via digital massage, Super Pinky rubber ball, or Honan intraocular pressure reducer [16]. In the case of tight lids, a lateral canthotomy and cantholysis can be performed [10]. In the case of a scleral prolapse, especially after PKP, a scleral support ring can prevent anterior scleral collapse [17]. Here, we describe a combination of a modified DALK over PKP to maintain a closed system and prevent PVP-related complications.

The study was institutional review board (IRB) exempt and adhered to the tenets of the Declaration of Helsinki.

## Technical report

**Figure 1:**
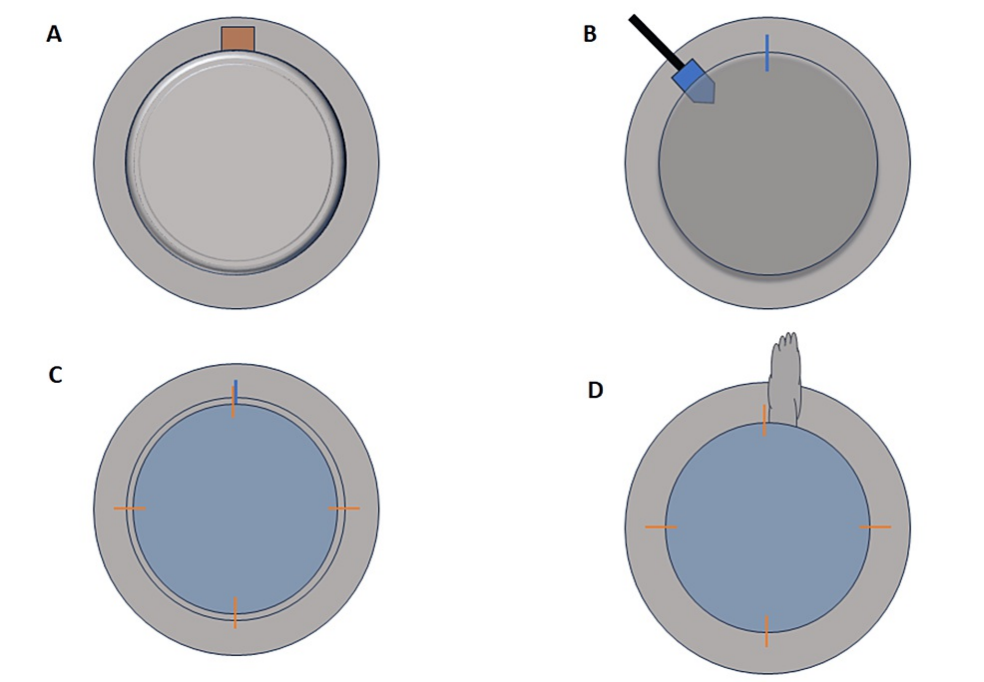
DALK-over-PKP technique (A): During trephination of the host cornea, the iris is found to prolapse due to elevated PVP. The superior aspect of the panels illustrate the inferior aspect of the eye (6 o’clock) in a patient lying supine. (B): After failed attempts at reducing PVP, the partially trephined cornea is sealed with a 9-0 nylon suture.  A diamond blade is then used to dissect down to the Descemet membrane performing a DALK. (C): Full-thickness donor cornea (in blue) is placed on top of the host Descemet membrane, and four 10-0 nylon cardinal sutures are placed through the donor cornea and host cornea outside of the optical zone (orange). The final 6 o’clock suture near the 9-0 suture placed earlier is not fully tightened. (D): The 9-0 suture along with the underlying host Descemet membrane is removed, followed by simultaneously tightening the 6 o'clock 10-0 nylon suture (orange). The case is then completed with 12 further sutures as is standard for PKP.

PKP begins in a standard manner with the measurement of the host cornea via calipers and recipient corneal trephination using a corneal trephine to match the host dimensions. This is followed by filling the anterior chamber with viscoelastic and trephination of the host cornea. In some cases, the beginning of the host trephination showed signs of PVP, such as iris prolapse (Figure 1A). At our institution, in the setting of PVP that does not respond to Super Pinky Ball pretreatment, loosening of the lid speculum, and lateral canthotomy, a modified DALK-over-PKP procedure was performed to maintain a closed anterior chamber and prevent any complications. Other preferred pressure-lowering methods may be employed as per institutional-specific protocol.

This modified technique begins after unresponsive PVP is confirmed following initial full-thickness penetration of the host cornea. After any uveal prolapse is resolved, the anterior chamber is reformed using viscoelastic, followed by a 9-0 nylon suture to partially close off the initial puncture, thus re-establishing a closed system. An opening is left part of the way through at the level of the stroma to provide an access point for the DALK. The host stroma and epithelium are manually dissected from the initial incision site using a 1 mm diamond blade with the “big-bubble” approach to visualize depth. Any other visualization method can also be used to ensure proper dissection (Figure 1B). Once fully separated, the overlying partial-thickness host cornea is removed while keeping the host Descemet membrane and endothelium in place. The full-thickness donor cornea is then placed on top of the host Descemet membrane, and cardinal sutures are placed at 12, 9, 6 and 3 o’clock using 10-0 nylon staying anterior to the host Descemet membrane. The 6 o’clock suture is not fully tightened. The original 6 o’clock 9-0 suture, which now only incorporates the host Descemet membrane and endothelium centrally, is then removed along with the incorporated central host tissue via the 6 o’clock position while simultaneously tightening the 6 o’clock 10-0 nylon suture (Figure 1D). The remaining interrupted sutures are then placed about the circumferential borders of the graft as is standard. The result is a PKP that was performed initially over a DALK to mitigate any risk of complications related to refractory PVP.

## Discussion

Despite the advantage of other partial-thickness corneal transplant techniques, PKP remains necessary for certain cases and pathologies. As a technique with so many potential complications, it is important to be able to have surgical techniques to respond to these potential intraoperative situations, such as PVP. One technique is to perform an anterior vitrectomy during a PKP and alternate cornea removal with vitreous removal [18]. Another group of techniques include the graft-over-host technique where the graft is sutured over the patient’s cornea to mitigate PVP, followed by complete removal using a quadrant-by-quadrant approach of the remaining host cornea [19,20].

While these graft-over-host techniques are similar to DALK-over-PKP, our technique maintains a true closed system by repairing the patient’s Descemet membrane with the initial 9-0 nylon suture while the other techniques use the increased pressure of the donor cornea to mitigate PVP. The graft-over-host technique thus leaves the potential for PVP-related complications to resurface during removal of each quadrant as the donor cornea is only secured via the 3, 6, 9 and 12 o’clock position sutures. In DALK-over-PKP, the main risk for PVP resurfacing is during the final removal of the Descemet membrane and endothelium. This can be mitigated by partially placing the 6 o’clock suture and tightening it as the remaining host tissues are removed. The other main limitation of DALK-over-PKP is the difficulty of the surgical technique and therefore the potential for damage to the overlying graft. A major limitation of outcomes in DALK and other partial thickness corneal transplant techniques is surgeon experience. Using this modified technique during a PKP, an inexperienced surgeon could create an opportunity to practice DALK in a real setting without worrying about damage to the underlying host Descemet membrane or endothelium as they will be discarded.

## Conclusions

Current mitigation strategies in the case of refractory PVP during PKP include pretreatment with external pressure, lateral canthotomy, and graft-over-host techniques. The existing graft-over-host techniques involve a full-thickness graft implanted over a donor cornea followed by quadrant-by-quadrant removal of host cornea, which may lead to resurfacing PVP. We describe the DALK-over-PKP technique as an alternative method to complete PKP while avoiding complications caused by PVP. This is accomplished by repairing the host Descemet membrane after initial perforation, performing a modified DALK and maintaining a true closed system throughout the procedure.
